# Single-Cell Sequencing Analysis of the db/db Mouse Hippocampus Reveals Cell-Type-Specific Insights Into the Pathobiology of Diabetes-Associated Cognitive Dysfunction

**DOI:** 10.3389/fendo.2022.891039

**Published:** 2022-06-01

**Authors:** Shizhan Ma, Wenkai Bi, Xueying Liu, Shangbin Li, Yaxin Qiu, Chengcheng Huang, Renjun Lv, Qingqing Yin

**Affiliations:** ^1^ Department of Endocrinology, Shandong Provincial Hospital Affiliated to Shandong First Medical University, Jinan, China; ^2^ Department of Geriatrics, Shandong Provincial Hospital Affiliated to Shandong First Medical University, Jinan, China; ^3^ Clinical Education Administration, Affiliated Hospital of Shandong University of Traditional Chinese Medicine, Jinan, China; ^4^ Department of Geriatric Neurology, Shandong Provincial Hospital Affiliated to Shandong First Medical University, Jinan, China

**Keywords:** diabetes-associated cognitive dysfunction, hippocampus, microglia, inflammation, single-cell transcriptomics

## Abstract

Diabetes-associated cognitive decline (DCD), is one of the complications of diabetes, which is characterized by a series of neurophysiological and pathological abnormalities. However, the exact pathogenesis of DCD is still unknown. Single-cell RNA sequencing (scRNA-seq) could discover unusual subpopulations, explore functional heterogeneity and identify signaling pathways and potential markers. The aim of this research was to provide deeper opinion into molecular and cellular changes underlying DCD, identify different cellular types of the diabetic mice hippocampus at single-cell level, and elucidate the factors mediating the pathogenesis of DCD. To elucidate cell specific gene expression changes in the hippocampus of diabetic encephalopathy. Single-cell RNA sequencing of hippocampus from db/m and db/db mice was carried out. Subclustering analysis was performed to further describe microglial cell subpopulations. Interestingly using immunohistochemistry, these findings were confirmed at the protein level. Single cell analysis yielded transcriptome data for 14621 hippocampal cells and defined 11 different cell types. Analysis of differentially expressed genes in the microglia compartments indicated that infection- and immune system process- associated terms, oxidative stress and inflammation play vital roles in the progression of DCD. Compared with db/m mouse, experiments at the protein level supported the activation of microglia, increased expression of inflammatory factors and oxidative stress damage in the hippocampus of db/db mouse. In addition, a major finding of our research was the subpopulation of microglia that express genes related to pro-inflammatory disease-associated microglia (DAM). Our research reveals pathological alterations of inflammation and oxidative stress mediated hippocampal damage in the db/db mice, and may provide potential diagnostic biomarkers and therapeutic interventions for DCD.

## Introduction

Metabolic diseases such as diabetes mellitus impairs the function of the brain and are called diabetic encephalopathy, including functional impairment of cognition, neuronal signal transduction, synaptic plasticity and neurophysiological changes, and potential structural damage associated with diabetes mellitus (1–3). Diabetes-associated cognitive dysfunction (DCD), a major complication of diabetes, is gaining more attention (4). The impairment of cognition in diabetes includes impaired learning, memory, problem−solving, attention and reduced information processing speed (1). Additionally, numerous clinical studies have shown that diabetes mellitus is closely related to vascular dementia, as well as Alzheimer’s disease (AD) (5, 6). Multiple studies have demonstrated that factors such as neuronal apoptosis, oxidative stress, neuroinflammation and altered neurogenesis may play a role in DCD (7, 8), in which oxidative stress and neuroinflammation are early-onset mechanism in diabetic encephalopathy (9, 10). The hippocampus plays a vital role in learning and memory, and the impairment of its function is linked to DCD (11). However, the mechanism by which diabetes impairs cognitive function have not yet been clearly established, including changes in these individual cellular compartments of the hippocampus and how they interact with each other.

Analysis of gene expression in hippocampal cells is an appropriate approach to decipher pathological changes in DCD, but there is a high degree of cellular heterogeneity in hippocampal cells. The current advent of scRNA-seq technology can discover new cell subpopulations and further explore gene regulatory mechanisms to reveal heterogeneity in genes and functions of each cell. Recent studies have shown that scRNA-seq in a mouse model of AD (5xFAD) identified a novel disease-associated microglia type (DAM), and revealed significant molecular heterogeneity within DAM, including pro-inflammatory and anti-inflammatory phenotypes (12, 13). Thus, this technology provides the opportunity to dissect different cell types in complex tissues, such as the hippocampus at single-cell resolution, which provides insights into the transcriptional signature in individual cells from a more detailed and microscopic perspective (14–16).

To explore the pathophysiology of DCD, we used db/db mouse, a model of type 2 diabetes (T2DM), characterized by the homozygous mice express deficient leptin receptors, which results in T2DM phenotypes including hyperglycemia, severe obesity, hyperphagia, polyuria and metabolic syndrome. In our present research, we applied scRNA-seq technology captures T2DM-induced gene changes in a large number of hippocampal cells and provides a comprehensive and detailed view of cell alterations occurring in the hippocampus to decipher the pathology of DCD.

## Materials and Methods

### Animals

Animal studies were performed in compliance with the requirements of the National Laboratory Animal Use Act of the People’s Republic of China and in accordance with protocols approved by the Animal Care and Use Committee of Shandong Provincial Hospital Affiliated to Shandong First Medical University. Twelve-week male db/db mice (BKS.Cg-Dock7m+/+ Leprdb/J, n=8) and db/m (Dock7m +/+ Leprdb, n=8) were obtained from Changzhou Cavens Laboratory Animal Co., Ltd (Jiangsu, China). They were kept in a temperature-controlled room (23 ± 1°C) under a 12 h light/dark cycle and allowed free access to chow and water. Animals with fasting plasma glucose levels >300 mg/dl were classified as diabetic.

### Morris Water Maze Test (MWM)

Using the MWM test, we assessed spatial learning and memory ability of eighteen-week mice (17). The maze apparatus includes a circular plastic pool filled with water added with black in kat approximately 22 ± 1°C. The circular plastic pool is divided into 4 quadrants, one of which includes an escape platform (diameter, 5 cm; height, 15 cm) was placed 1 cm underwater and at a fixed position. The test composed of a 5-day acquisition phase trial and a probe trail on day 6. During acquisition phase trials, the animals were placed in the water, were able to reach to the platform within 60 s, which were remained on the platform for 10 s, and each animal was trained four times every day. However, if the mice failed to find the platform within 60 s, they were gently guided to the platform and remained there for 10 s, and the escape latency was recorded as 60 s. On the sixth day, the spatial exploration test with the platform removed was carried out, and rats were permitted to swim for 60 s. Data of the time spent and the number of platform crossings in the target quadrant within 60 s were recorded. The mice movement of the escape latency was recorded using a computerized video system and analyzed by a computer system.

### Hippocampus Dissection and Dissociation

The protocol for dissection and single-cell dissociation were performed as previously described (18). After Morris Water Maze testing was completed, mice were immediately euthanized with sodium pentobarbital (50 mg/kg, i.p.) and systemically perfused with cell culture grade saline (0.9%, sigma). The brains were removed and samples of the hippocampus of the brain were immediately isolated from the ipsilateral side of the brain on ice, which dissociated into a viable single-cell suspension. Briefly, using scalpels, we mince hippocampus samples, which further were digested in Earle’s Balanced Salt Solution (EBSS) containing DNAse I (0.01 mg/ml) and papain (1 mg/ml) for 60 minutes at 37°C. Hyaluronidase prevents dissociation. Intact cells were isolated on a single step discontinuous density gradient and resuspended in phosphate buffered saline containing 0.04% weight/volume bovine serum albumin (BSA), for single cell transcriptomic analysis processing.

### ScRNA-Seq

Hippocampus single-cell suspensions from db/db and db/m mice were added to the Chromium Single Cell Controller Instrument (10x Genomics, Shanghai Genechem Co.,Ltd.) to create single-cell gel beads. According to the manufacturer’s protocol, the scRNA-seq libraries were prepared with the Chromium Single-cell 3′ Reagent V3 Kits, and sequenced on an Illumina HiSeq X Ten System. We obtained 150 bp paired-end reads.

### ScRNA-Seq Data Analysis

Raw sequencing data, cellular barcodes were demultiplexed by using Cell Ranger Software Pipeline (version 3.0), using the STAR aligner (version 3.1.0), we map reads to the mouse reference genome and transcriptome. To produce a matrix of gene counts versus cells, down-sample of reads was required to produce normalized aggregate data across samples. The unique molecular identifier (UMI) count matrix was processed by using R package Seurat (version 3.1.1) (19). In order to eliminate low-quality cells and likely multiplet captures, the cells with >200 genes and <8000 genes; >400 UMIs and <20% mitochondrial RNA (mtRNA) were retained for subsequent analyses. The filtered matrix was normalized for library size to gain normalized counts in Seurat.

Identification of the top variable genes in individual cells was performed (20). In summary, principal component analysis (PCA) was performed on the highly variable genes to lessen the dimensionality, and the top principal components (PCs) were chosen for cell clustering using a graph-based clustering method. We visualised the clustering results using t-Distributed Stochastic Neighbor Embedding (t-SNE) technique. t-SNE dimensional reduction was performed in R using the Seurat package. Compared with classical cell type markers, the identified 26 cell clusters correspond to 10 cell types. In order to quantify changes in hippocampal intercellular communication during diabetes mellitus, CellPhoneDB (V2.0) was used to identify biologically relevant ligand receptor partners (21). To characterize intercellular correspondence organizations, we connected any two cell types in which ligands and receptors were communicated in the previous and last cell types, separately.

The Find Markers function (test.use = bimod) of Seurat package was used to identify differentially expressed genes. P-value < 0.05 and |log2foldchange | > 0.58 were set as the differential expression thresholds. Differentially gene expression (DEGs) was subjected to GO enrichment and KEGG pathway enrichment analysis using hypergeometric distribution in R package, and functional association networks were constructed and defined using STRING version 11.0.

### Immunofluorescence Staining

PFA fixed brain tissues were cut into cryosections. Nrf2, HO-1, Nlrp3, Iba-1, Trem2 and Cmklr1 expression levels in brain tissue sections were characterized using immunofluorescence.

Cryosections were air dried at room temperature, rinsed with PBS, supplemented with 0.4% Triton X-100, and submitted for antigen retrieval. Sections were blocked with 10% normal goat serum for 1 h at 37°C. The sections were incubated with anti-Nrf2 (GB113808, Abcam; 1:500), anti-HO-1 (GB11845, Abcam; 1:200), anti-NLRP3(GB11300, Abcam; 1:200), anti-TREM2 (bs-2723R, Abcam; 1:200), anti- Cmklr1 (bs-10185R, Abcam; 1:200) or anti-Iba-1 (GB12105, Abcam; 1:500) at 4°C overnight. The next day, sections were washed and incubated with tetramethylrhodamine labeled anti mouse IgG for 1 h at room temperature; Nuclei were dyed with 4, 6- diaminido-2-phenylindole (DAPI) before 10 min of mounting. Sections were then analyzed under a laser scanning confocal microscope (Olympus, Tokyo, Japan). We analyzed the images using Image-Pro Plus 6.0 software (Media Cybernetics, USA). The results were analyzed using GraphPad Prism 5.0 software and are showed as the mean ± standard error of mean. Statistically significant differences were determined using a two-tailed unpaired Student’s t test. P<0.05 was considered statistically significant. A two-tailed unpaired Student’s t-test was used to determine statistically significant differences. P < 0.05 was considered statistically significant.

## Results

### Impairments of Learning and Memory In db/db Mice

**Figure 1:**
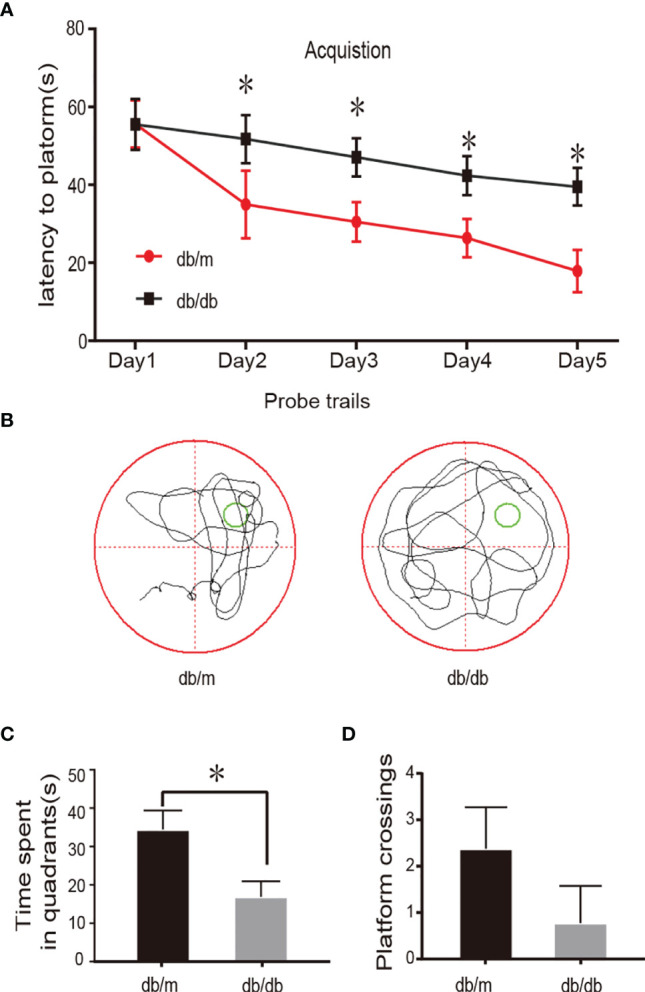
Learning and memory impairment in db/db mouse. In the MWM test, day 1 represents performance on the first trial, and subsequent scores show average of all daily trials. **(A)** Escape latency of the 5-day acquisition trials in MWM. **(B)** Representative swim pathways of respective groups in the spatial probe test. **(C)** Time spent in the target quadrant during the probe trial. **(D)** The numbers of the target platform crossings in the probe trial. Data are represented mean ± SEM for 3 mice in each group. ^⁎^ *P* < 0.05 vs. db/m group.

The Morris water maze test is commonly used to study spatial learning and memory loss in animals. During the acquisition phase trials, escape latencies did not differ significantly between groups on day 1 (P> 0.05; **Figure 1A**), in addition, the time to find the hidden platform was declined progressively from the second day to the fifth day. Interestingly, the db/db group spent significantly more time finding the hidden platform compared to the db/m group (P < 0.05; **Figure 1A**). The typical swimming traces of mice is illustrated in **Figure 1B**, the db/db mouse showed a more disorganized and longer swimming paths. The hidden platform was removed during the spatial probe trial. As expected, compared with that of the db/m group, the time spent in the target quadrant of db/db group and the number of platform crossings were was markedly reduced (P < 0.05; **Figures 1C, D**). In summary, these results based on the MWM test demonstrated impaired learning and memory in db/db mice. In the current study, all db/db mice developed concomitant cognitive decline. Hence, we performed single-cell transcriptome analysis of hippocampal tissues from db/m mice and db/db mice.

### ScRNA-Seq Analysis Resulted in 26 Clusters and Identified Ten Cell Types

**Figure 2:**
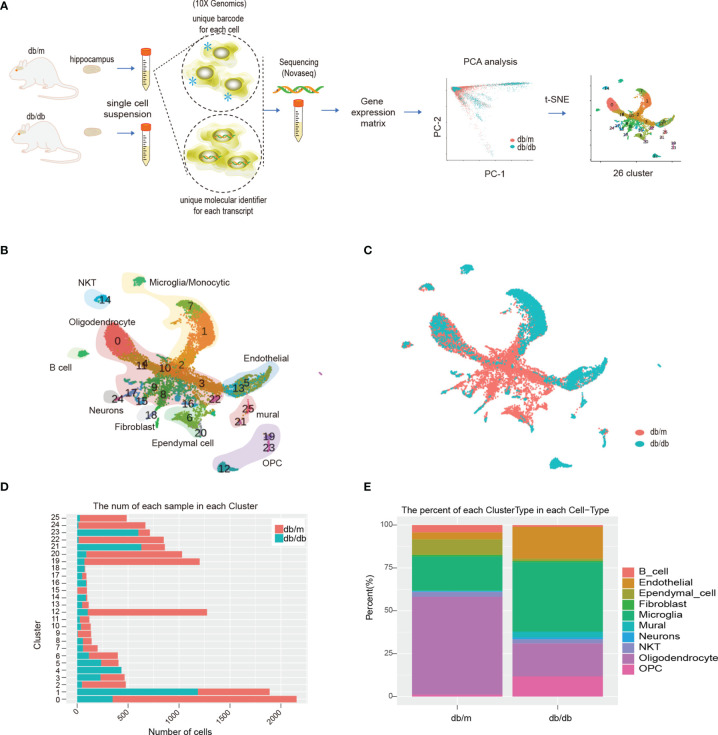
Schematic diagram of the experimental workflow, and scRNA-seq analysis of db/db vs db/m mouse hippocampus. The workflow is illustrated in **(A)**, * represent barcode sequence labeled cells. **(B)** t-SNE plot of 14621 cells isolated from db/db and db/m hippocampus, exhibiting 26 clusters that were identified. **(C)** t-SNE plot of 14621 cells isolated from the db/db and db/m hippocampus, colour coded according to genotype. **(D)** Number of db/db (orange) and db/m (cyan) hippocampus cells per cluster. **(E)** Relative abundances of the different hippocampus cell types per genotype.

Isolated hippocampus from db/m and db/db mouse were separated into single-cell suspensions. After quality control filters, A total of 14621 cells were used for a following analysis. Of these, 4667 cells (32%) originated from db/db mice and 9954 cells (68%) from db/m mice. After the completion of quality control, PCA and t-SNE analysis was conducted. The workflow of this study is shown in **Figure 2A**. Based on expression of well-established markers, the cells were classified into 26 transcriptionally distinct clusters (**Figures 2B–E**), and divided into 10 distinct cell types based on cell-type-specific gene expression and annotated as follows: B cells, Endothelial cells, Ependymal cells, Fibroblast, Microglia, Mural cells, Neurons, NKT, Oligodendrocyte, Oligodendrocyte Precursor Cell (OPC). The abundance of db/m vs db/db cells per cluster is represented in **Figures 2C–E** shows the relative abundance of cell types for each genotype. In this scRNA-Seq data, compared with db/m mice, the relative abundance of microglia and oligodendrocyte in db/db mice changed significantly (**Figure 2E**), so we first analyzed this type of cells, and further performed DEGs and/or sub cluster analysis on these data.

### DEGs Analysis in db/db vs db/dm Mice Microglia

**Figure 3:**
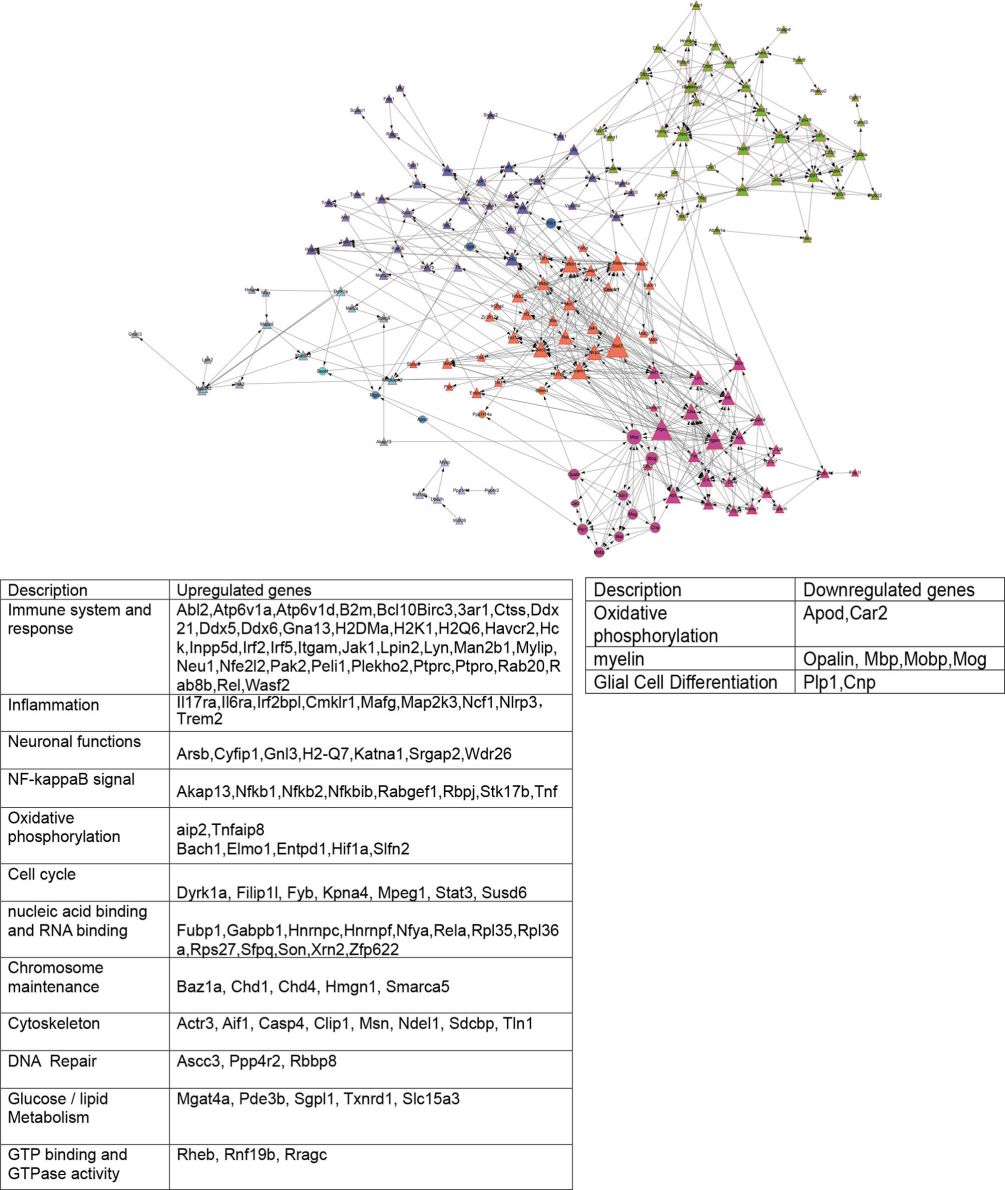
STRING analysis of DEGs networks in db/db with db/m microglia. Shapes represent up - and downregulated messages. Triangles represent upregulated gene networks and circles represent downregulated gene networks; Different colors represent different cluster, the size of the dots represents the degree.

**Figure 4:**
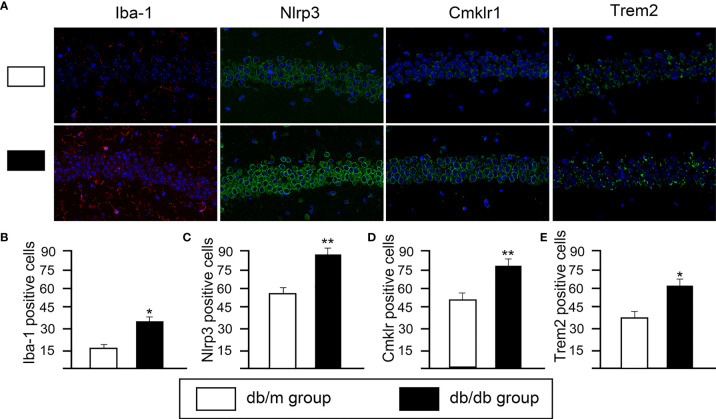
The changes of microglial activation and neuroinflammation in hippocampal CA1 sections from from db/m and db/db group. **(A)** Representative immunofluorescence staining of Iba-1, Nlrp3, Cmklr1 and Trem2. Representative images of Iba-1 (red) staining and representative images of Nlrp3, Cmklr1, Trem2 (green) staining. Nuclei are stained with DAPI (blue). (scale bar = 50 mm). Quantification of Iba1- **(B)**, Nlrp3- **(C)**, Cmklr1- **(D)**, Trem2- **(E)** positive cells in the db/db mice hippocampus. All data are represented as means ± S.E.M for 8 mice in each group. *P < 0.05, **P < 0.01 vs. db/m group.

**Figure 5:**
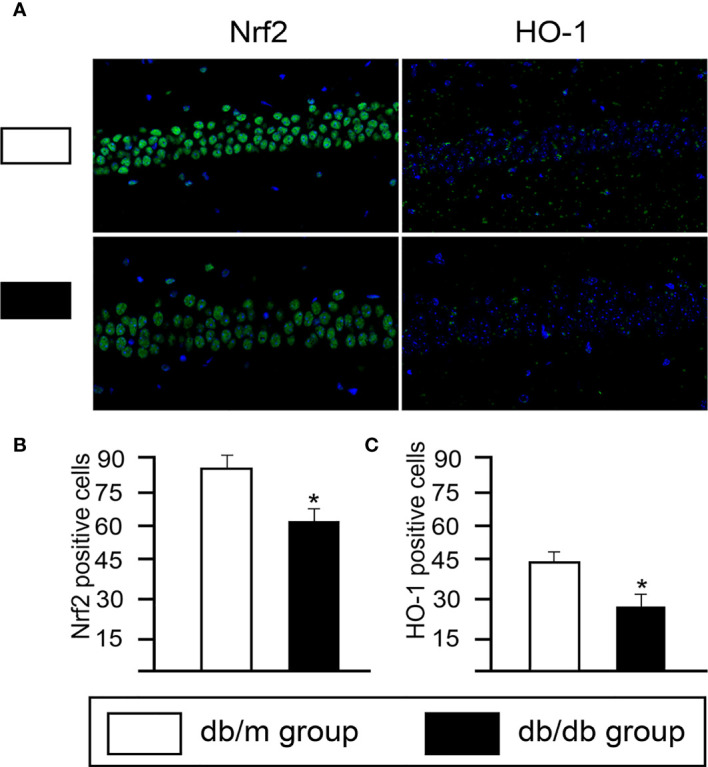
The changes of expression of Nrf2/HO-1 system in hippocampal CA1 sections from from db/m and db/db group. **(A)** Immunofluorescence analysis of Nrf2 and HO-1. Representative images of Nrf2 and HO-1 (green) staining in the hippocampus. Nuclei are stained with DAPI (blue). (scale bar = 50 μm). Quantification of Nrf2- **(B)** and HO-1- **(C)** positive cells in the db/db mice hippocampus. All data are represented as means ± S.E.M for 8 mice in each group. *P < 0.05 vs. db/m group.

Based on DEGs, the following functional networks upregulation were found in db/db microglia (**Figure 3**; **Supplementary Table 1**): immune system and response, inflammation, neuronal functions, OXPHOS, Cell cycle, NF-κB signal, nucleic acid binding and RNA binding, chromosome maintenance, cytoskeleton, DNA Repair, glucose/lipid metabolism, GTP binding and GTPase activity. Notably, microglia are immune cells that participate in inflammatory responses in central nervous system (CNS), we observed several markers of inflammatory response, including Il7ra, Il6ra, Irf2bpl, Cmklr1, Mafg, Map2k3, Ncf1, Nlrp3 and Trem2. To determine glial activation and inflammation factors, immunofluorescent staining for in the hippocampus of mice were performed. Iba-1 is highly expressed in activated microglia. We found that the expression of Nlrp3, Cmklr1, compared with db/dm group, the number of positive cells labeled by TREM2 and Iba-1 in hippocampus of db/db group was significantly increased (**Figure 4**), suggesting that T2DM facilitates microglial activation and increases levels of inflammatory factors in the hippocampus. Additionally, Immunofluorescence was also used to analyze the expression of Nrf2/HO-1 system, which has beneficial effects by protecting against oxidative damage (22). As seen from **Figure 5**, the db/db group rats showed significantly lower levels of Nrf2 and HO-1 in the hippocampus (P<0.05) compared to the db/m group rats, suggesting the antioxidant system was damaged in the db/db mice. Downregulated genes in db/db mice microglia associated into OXPHOS, myelin and glial cell differentiation (**Figure 3**; **Supplementary Table 1**).

**Figure 6:**
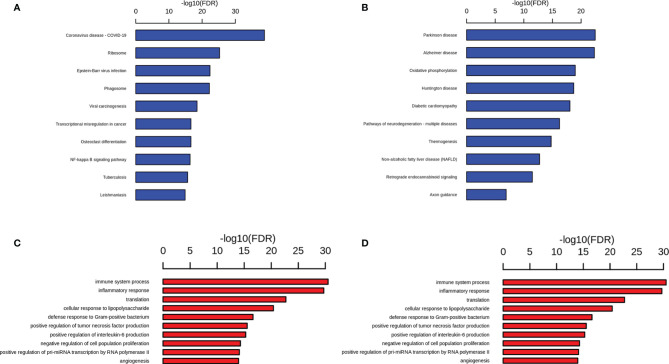
DEGs analysis reveal altered pathways in microglial cells in db/db mice. Representative GO **(A)** and KEGG **(C)** terms of upregulated gene in db/db mice microglial cells versus db/m microglial cells and representative GO **(B)** and KEGG **(D)** terms of downregulated gene in db/db mice microglial cells versus db/m microglial cells. Significance is indicated as a *P* value calculated using the Fisher exact test (*P*< 0.05) and expressed as -log_10_ (*P* value).

Compared with the db/dm mice microglia, 768 differential genes were expressed in the db/db mice microglia, of which 386 genes were up-regulated and 382 genes were down-regulated (**Supplementary Table 2**). Functional enrichment analysis of upregulated genes was performed using the Kyoto Encyclopedia of Genes and Genomes (KEGG). The top 10 most remarkably enriched KEGG pathways were exhibited in microglia. KEGG pathways analysis suggested that compared with the db/dm mice microglia, the enrichment items of the db/db mice microglia were mainly concentrated in infection- and inflammation-associated pathways, such as pathways in Coronavirus disease-COVID-19, Epstein-Barr virus infection, phagosome and NF-κB signaling pathway (**Figure 6A**; **Supplementary Table 3**). Previous studies demonstrated that brain microglial overactivation can induce proinflammatory gene expression by activation of NF-κB signaling pathway following stroke (23), The down-regulated genes were evaluated by KEGG analysis, and the KEGG pathways were mainly enriched for terms involved in Parkinson disease, AD, oxidative phosphorylation, Huntington disease and Pathways of neurodegeneration-multiple diseases (**Figure 6B**; **Supplementary Table 4**). GO term analysis of the upregulated genes of microglia revealed enrichment of expected biological processes such as immune system process, inflammatory response, cellular response to lipopolysaccharide, defense response to Gram−positive bacterium and positive regulation of interleukin-6 (**Figure 6C**), and it has also been reported that microglial inflammatory activation, stimulated by diabetes mellitus (DM), which caused memory deficits, gene alterations in brain endothelium (24). Furthermore, GO analysis was performed on the down-regulated genes (fold change > 1.5), and the gene functions were primarily enriched in the items related to myelination, microtubule cytoskeleton organization, neuron projection development, nervous system development and oligodendrocyte differentiation. (**Figure 6D**). Consistent with our findings, microglial plays a crucial role in neurodegeneration, probably as a result of the different pathological substrates associated with neurodegenerative diseases as well as systemic inflammation and factors influencing insulin resistance, such as T2DM (25). Taken together, the results of GO and KEGG analysis reveal that the T2DM could induce the activation of microglia which in turn might cause learning and memory, as well as an increased risk of neurodegenerative diseases.

### Diverse Microglial Activation Responses Are Triggered in db/db Mice

**Figure 7:**
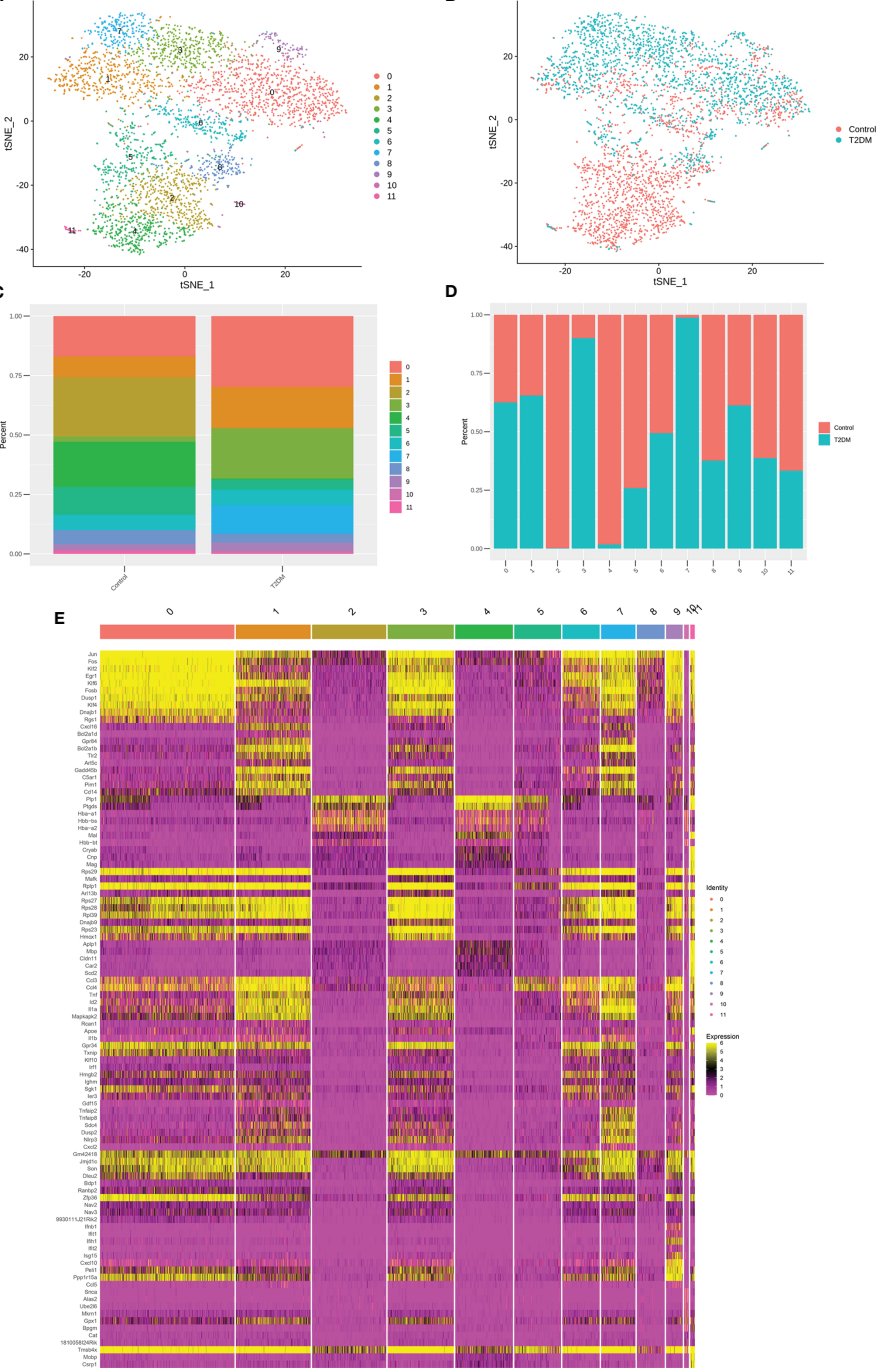
Subclustering of microglial cells. **(A)** t-SNE plot showing microglial cells subclusters. **(B)** t-SNE plot of microglial cell, colour coded according to genotype. **(C)** Percentage of cells in each microglial cell cluster. **(D)** Cell cluster percentages across two experimental groups (db/db and db/m). **(E)** The heatmap showing the expression levels of specific genes in microglial cells subclusters.

Study has often referred to the response of microglia to injury or pathology as ‘‘activated,’’ an umbrella term that refers to biochemical and physiological deviations from homeostasis. Activated microglia are observed in nearly all neurological disorders, including neurodevelopmental and neurodegenerative diseases. However, it is unclear whether or how microglia regulate their response to specific types of injury (T2DM induced brain injury). Next, we assessed whether microglial cells in our data could be subdivided into subpopulations based on distinct activation states, cell markers and biological pathways and determined twelve subclusters with a microglial transcriptional profile (**Figures 7A, B**; **Supplementary Table 5**). Subsequently, we sought to determine the proportion of each subpopulation (**Figures 7C, D**), and perform DEGs and gene set enrichment analysis. Interestingly, subpopulation 0, db/db mice had an increased proportion of inflammatory related subpopulations of microglia, such as subpopulation 1, subpopulation 3, subpopulation 7 and subpopulation 9 (**Figure 7C, D**). We identified the marker genes [sorted by average log2(fold change)] for each subpopulation relative to all other microglia subpopulations, and these genes were plotted using a heatmap (**Figure 7E**; **Supplementary Table 6**).

**Figure 8:**
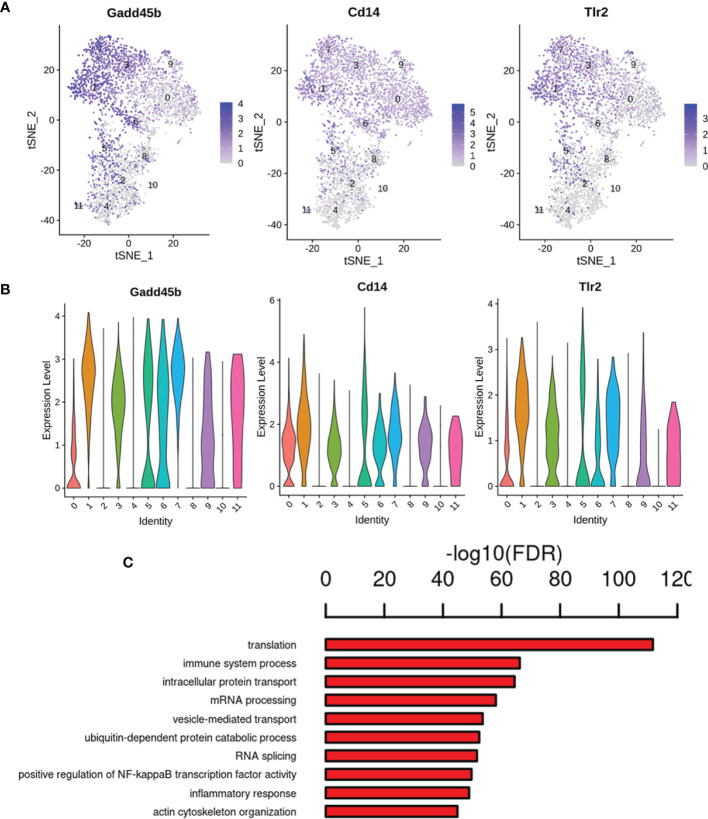
Gene expression pattern analyses in cluster 1. **(A)** Feature plot of inflammation marker genes Gadd45b, Cd14 and Tlr2 in cluster 1. **(B)** Violin plots revealed the expression of inflammation marker genes for 1 cluster. **(C)** The enriched GO terms of cluster 1 are shown.

Our gene expression analysis revealed that most of the analyzed cells highly expressed marker microglia genes (C1qa, Fcrls, Cx3cr1, P2ry12/P2ry13, and Trem2) (26), but not all cells (**Supplementary Table 6**), indicating that existing detection techniques and marker definitions require further exploration. Except for the highly expressed marker genes, genes significantly upregulated were also found in specific microglial states, such as AD risk genes (**Supplementary Table 6**) (Ctsb, Ctsd, Trem2, and Tyrobp) are enriched in the activated response microglia (ARM), which was identified main activated microglia states by Sala Frigerio et al. (26). This highlights cognitive dysfunction in diabetes and the pathogenesis of AD may share some commonalities. We also observed upregulation of neurodegeneration- and inflammation- related genes such as Csf2ra, Jun, Cox6c, Tlr7, and Ttr. We found that cluster 1 expressed the higher levels of Gadd45b (Growth arrest and DNA-damage-inducible 45 beta) gene, which acts as an apoptosis factor and mediator of neuroinflammation (13, 27, 28), and inflammation makers (Cd14 and Tlr2) (13, 29), and showed enrichment for the GO term regulation of immune system process, inflammatory response and positive regulation of NF-κB transcription factor activity (**Figures 8A–C**). Additionally, the cluster 10 expressed the highest levels of the two best-known inflammation genes, Gpx1 and Cat (30) (**Supplementary Figures 1A–D**), the GO terms, including hydrogen peroxide catabolic process, heme biosynthetic process, response to oxidative stress and response to hydrogen peroxide (**Supplementary Figure 1E**). It is well known that persistent hyperglycemia causes oxidative stress, which leads to brain damage (31).

**Figure 9:**
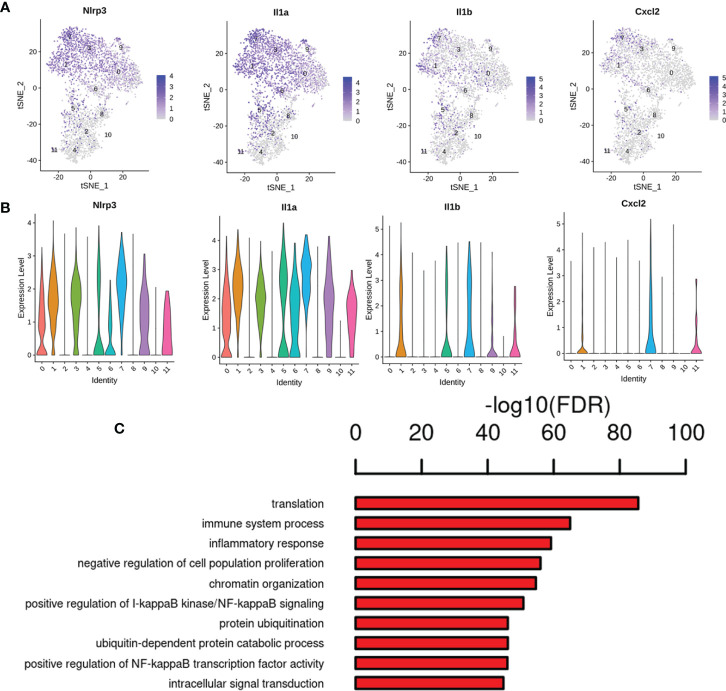
Gene expression pattern analyses in cluster 7. **(A)** Feature plot of inflammation marker genes Nlrp3, Il1a, Il1b and Cxcl2 in cluster 7. **(B)** Violin plots revealed the expression of inflammation marker genes for 1 cluster. **(C)** The enriched GO terms of cluster 7 are shown.

Interestingly, increased transcripts were observed for other Ms4a family members (Ms4a6b, Ms4a6d, Tmem176a, and Tmem176b) expressed by microglia (Cluster 0, 1, 3, 5, 6, 8, 9) (**Supplementary Table 6**). Genes such as macrophage migration inhibitory factor (Mif), lactate dehydrogenase A (Ldha), and triosephosphate isomerase 1 (Tpi1) were found to be upregulated in cluster 5 microglia (**Supplementary Table 6**). Of note, we found the most significantly enriched genes in cluster 7 microglia expressed many inflammatory signals that were not normally expressed in other microglial clusters (**Figures 9A-B**; **Supplementary Table 6**). They upregulated various pro-inflammatory chemokines Nlrp3, Lgals3, Cxcl2, Cxcl10, Cxcl16 and inflammatory cytokine interleukin 1 (Il1a, Il1b), as well as Ccl4 and Ccl3 (32, 33). The ligand for chemokine receptor type 5 (Ccr5) is Ccl4, also referred to as macrophage inflammatory protein-1β (Mip-1β), which adjusts the transmission and responder functions of different immune cells (34). Additionally, cluster 7 showed enrichment for the GO term regulation of immune system process, inflammatory response, positive regulation of I-κb kinase/NF-κB signaling and NF-κB transcription factor activity (**Figure 9C**), leading us to postulate that these cells represent pro-inflammatory lesional microglia, which might be defined as into the phenotypes M1 (proinflammatory) (35). Recently Rangaraju et al. found significant molecular heterogeneity in DAM (disease-associated microglia) (13). Interestingly, genes related to pro-inflammatory DAM, such as Tlr2, Ptgs2, Il1b and regulators (NF-κb, Stat, and RelA), were mainly enriched in cluster 7 microglia (**Supplementary Table 6**). Moreover, cluster 6 microglia also expressed upregulated the inflammatory signals genes in cluster 7 microglia, indicating an overlap in transcriptional signaling and may be some association between these two states. These findings could be translated to human disease, and novel microglial pathogenic markers might serve as biomarkers or therapeutic targets, which also help to further define how we distinguish microglial activation states *in vivo*.

## Discussion

Cognitive impairment is an important complication in the CNS of diabetic patients, which could reflect diabetes induced changes in the brain. There are some studies suggesting the link between AD and T2DM, such as hyperglycemia, leading to glutamate induced excitotoxicity in neuronal cells and may contribute to oxidative stress, amyloid- β accumulation, tau hyperphosphorylation, formation of advanced glycation end products (36). Whereas, the multifactorial pathogenesis of diabetic encephalopathy remains unclear. The diabetic db/db mouse is a ideal animal model to study the pathophysiology of diabetes mellitus, because this organism, like humans, develops diabetes and its associated complications, including oxidative stress, obesity, and hyperglycemia. Furthermore, db/db mouse shows neurobehavioral deficits, autonomic behavior and memory impairment are important roles of hippocampal damage caused by T2DM (37). In the present study, our MWM results suggested that db/db mice suffered from impaired in memory ability and spatial learning, indicating db/db mice had deficits in long-term and spatial memory functions (38, 39). The complexity of hippocampus structural makes it vulnerable to a variety of pathological conditions such as diabetes and is one of the most sensitive regions of the brain to microenvironmental changes (1). Next, we explored the different cell types and their unique transcriptional features in the mouse hippocampus at single-cell level using scRNA-seq technology. Overall, the results of analysis based on changes in cell abundance and intercellular communication suggest different hippocampus cells exhibit different degrees of sensitivity to DCD. Analysis of DEGs between the db/m and db/db groups showed that gene transcription in each cell type underwent widespread changes in db/db mice.

The pathogenesis of DCD is complex and not very clear, among which the more definite view is that persistent inflammation caused by the secretion of a large number of pro-inflammatory factors and pro-oxidant substances is the main contributing factor to DCD (40). Activated microglia of the CNS are major cellular responders to injury or infection, which may drive or perpetuate CNS inflammation by increasing the expression of inflammatory molecules, thereby exacerbating some of these detrimental processes (41). GO and KEGG pathway analyses showed that inflammation-, immunity-, and infection-associated terms were enriched in db/db mice microglia, such as Il17ra, Il6ra, Cmklr1, Nlrp3, Trem2, Coronavirus disease-COVID-19, Epstein-Barr virus infection, phagosome and NF-κB signaling pathway. Among immune cells in the CNS, which has a vital role in propagating neuroinflammation (42). Numerous evidences suggested that the activation of Nlrp3 plays a vital role in DCD and other neurodegenerative diseases (43, 44). Nlrp3 is mainly involved in neuroinflammation *in vivo* and *in vitro*. For example, recent studies have reported that Nlrp3 can activate Tlr4 signaling, leading to the neuroinflammatory responses (45), and illustrated the possible mechanism related to Nlrp3 inflammasome and peripheral inflammation in microglia of mice, and the aggravation of diabetes neuroinflammation in the cortex and hippocampus (46, 47). Additionally, research shows that the chemokine-like receptor 1 (Cmklr1) is a chemokine like G protein-coupled receptor expressed on specific cell populations in mice and humans, including inflammatory mediators, dendritic cells, neurons and microglia, regulating chemotaxis to the sites of inflammation and activation state (48, 49). Lately, it has been reported that Cmklr1 axis contributed to the development of diabetic cardiomyopathy on inflammation, which was primarily mediated by Nlrp3 inflammasome (50). Triggering receptor expressed on myeloid cells 2 (Trem2) is a single pass transmembrane receptor that activates a range of signaling pathways associated with immune function through ligand binding (51). Trem2 has been used as a microglial activation marker and a general requirement for its activation (29, 52). Galectin-3 can bind to the Trem2, activate microglia, and induce neuroinflammatory responses in an AD mouse model, our recent study confirmed that inhibition of endogenous Trem2 ligand ameliorates DCD by inhibiting oxidative stress and neuroinflammation (53). In addition, studies have shown that hyperglycemia leads to NF‐κB activation and triggering the release of proinflammatory cytokines (54). Cytokines (IL and TNF‐α) expressed and secreted by microglia in the CNS and are involved in the regulation of neuroimmune endocrine networks (55). The histological features of many neurological diseases are mostly characterized as ‘‘microgliosis’’, which includes alterations in microglial morphology, change of gene expression, but also migration, growth and proliferation in response to injury (56). Iba-1, a microglia specific marker, has been reported to be widely used for microglial detection, and increased expression of Iba-1 could suggest microglial activation (57), which was also confirmed by our Iba-1 immunofluorescent staining results. In addition, Microglial activation was closely associated with neurodegenerative diseases, such as AD, Parkinson’s disease, and multiple sclerosis (40). Interestingly, our KEGG pathway analyses revealed that Parkinson disease, AD, Huntington disease and Pathways of neurodegeneration-multiple diseases were enriched in microglia.

The state of microglia in the CNS changes rapidly with the change of environment, but its molecular and functional characteristics are not clear (41). Accumulating evidence suggests that CNS microglial activation is heterogeneous, depending on the factors that microglia become activated, there are three states of microglia, “classical activation,” “alternative activation,” and “acquired deactivation” (58). Classical activation is related to the production of pro-inflammatory cytokines such as TNF-α, nitric oxide (NO) and proteases and are also called “M1 microglia,”, whereas alternative activation and acquired deactivation are termed “M2 microglia” (58). In brief, the M1 activation state is defined as the pro-inflammatory M1 phenotypes, and the M2 activation state is considered to anti-inflammatory or neuroprotective M2 phenotypes. In our results, compared with db/m mice, the relative abundance of microglia in db/db mice changed significantly, further analysis of differentially expressed genes revealed, the inflammation related genes were found to be upregulated in db/db microglia, which leading us to postulate that these cells represent pro-inflammatory lesional microglia, which might be defined as into the phenotypes M1 (proinflammatory). However, simple classification scheme might lump together heterogenous sets of microglia, the information obtained from histological research might be limited or even misunderstood.

Next, we identified twelve microglial subpopulations according to microglia with different activation states/biological pathways, which possessed unique molecular signatures in response to injury. We found that cluster 1 expressed the higher levels of Gadd45b gene, which was a member of the gene family related to apoptosis, and DNA damage repair (13). Studies have demonstrated that Gadd45b might be critically involved in neuroinflammation related neurological diseases (59, 60). Interestingly, our previous study confirmed Gadd45b modulated hippocampal neuroinflammation in animal model of post-stroke depression (PSD), Gadd45b may have therapeutic potential for CNS diseases through neuroinflammation (27). Of note, we found that inflammation associated terms were enriched in cluster 7, and express Nlrp3, Lgals3, Cxcl2, Cxcl10, Cxcl16 and inflammatory cytokine interleukin 1 (Il1a, Il1b), as well as chemokines Ccl4 and Ccl3. Additionally, regulation of immune system process, inflammatory response, positive regulation of I-κb kinase/NF-κB signaling and NF-κB transcription factor activity were enriched in cluster 7. We speculate that they might be a specialized group that uniquely initiates the inflammatory response. For example, Il1b can cause neurotoxicity (61), chemokines Ccl4 attracts infiltrating immune cells can exacerbate pathology. Lgals3 is the gene of galectin-3, our recent study showed that serum galectin-3 levels were remarkably increased not only in the of T2DM patients with mild cognitive impairment (MCI), but also in the serum and brain of T2DM mice, and we also confirmed galectin-3 was associated with neuroinflammation, oxidative stress, impaired learning and memory (53, 62). Our results suggested that galectin-3 could be a promising therapeutic target candidate for treating DCD. The functions of Ms4a family genes found in cluster (0, 1, 3, 5, 6, 8, 9) are currently not well defined and are primarily are transmembrane chemosensors (63), some of which regulate immune cell functions (64). Ms4a family members are a key modulator of Alzheimer’s disease risk (65), nevertheless, the functions of Ms4a family members in the disease is not understood. In addition, Mif in cluster 5 microglia is closely linked to the growth, motility, inflammation and immune regulation of immune cells in the CNS. Ldha and Tpi1 were related to glycolysis, indicating that the metabolic profile of these cells has changed. A change from oxidative phosphorylation to glycolysis and metabolism occurs when microglia are stimulated by adverse factors (40), and they regulate glucose metabolic profile after the transition to the active state, presumably to meet increased energy requirements.

A potential subtype of protective microglia, disease-associated microglia (DAM) were recently proposed in mice model of AD (5xFAD), and decipher their dynamics during AD progression (12). Interestingly, the conversion of microglia from a stable state to an activation status is thought to be a continual process, which includes two phases. The Trem2 independent stage (DAM1), involving activation of Tyrobp, Apoe, and B2m, and downregulation of the Cx3cr1 and P2ry12/P2ry13 genes, followed by the Trem2 dependent stage (DAM2) including upregulation of Cst7, Lpl, and CD9 genes (12). Research showed that DAM mitigates the disease through enhancing phagocytosis in the late stage of AD (12). In addition, single-cell analysis confirmed DAM have been identified in normal aging and in models of neurodegenerative disease, suggesting that DAM represent a general response to neurodegenerative diseases (66). Interestingly, we did not detect protective microglia associated with neurodegeneration in diabetes. DCD may be an early stage of dementia with MCI, or cognitive impairment due to diabetes related cerebrovascular damage could serve as an explanation. Whereas, our study found pro-inflammatory DAM emerges in hippocampus of T2DM mouse model and are characterized by expression of pro-inflammatory genes (Tlr2, Ptgs2, and Il1b) and regulators (NF-κB, Stat, and RelA) (13). Over all, our study discovers previously unknown heterogeneity of microglia, a potential detrimental microglia type associated with DCD. Additionally, understanding the heterogeneity within the DAM may find new biological opinions into diversity of microglia and might contribute to the discovery of immunomodulatory therapeutic targets and drugs for DCD.

## Conclusion

In conclusion, through single-cell RNA sequencing, we gained a deeper insight into the heterogeneity of microglial cell populations in the db/db mice hippocampus. We observed changes in the abundance of hippocampal cell populations, most notably microglial cell populations, which confirm the novel opinion that microglia play a vital role in DCD. Our findings in this article should be verified in additional early/intermediate stage DCD models, and to determine their relevance in humans, in patient samples. Moreover, further evaluation of the differentially expressed pathways in functional experiments is required to assess whether they play a deleterious or protective role in DCD.

## Data Availability Statement

The original contributions presented in the study are publicly available. This data can be found here: NCBI, GSE201644.

## Ethics Statement

The animal study was reviewed and approved by Ethics Committee of Shandong Provincial Hospital Affiliated to Shandong First Medical University.

## Author Contributions

SM, RL, QY, and WB designed the experiments, analysis was carried out by XL, SL, YQ, and CH. SM and QY supervised the study. RL and QY wrote the article. All authors contributed substantially to the discussion of the content and reviewed or edited the manuscript before submission.

## Funding

This work was supported by National Natural Science Foundation of China (Grant No.82000771), Shandong Provincial Natural Science Foundation (Grant No. ZR2021MH014, ZR2019PH017), and Jinan Municipal Science and Technology Project (grant no. 202134033).

## Conflict of Interest

The authors declare that the research was conducted in the absence of any commercial or financial relationships that could be construed as a potential conflict of interest.

## Publisher’s Note

All claims expressed in this article are solely those of the authors and do not necessarily represent those of their affiliated organizations, or those of the publisher, the editors and the reviewers. Any product that may be evaluated in this article, or claim that may be made by its manufacturer, is not guaranteed or endorsed by the publisher.

## Supplementary Material

Supplementary Figure 1 Gene expression pattern analyses in cluster 10. Feature plot of inflammation marker genes Gpx1 **(A)** and Cat **(C)** in cluster 10. Violin plots showing the expression of inflammation marker genes Gpx1 **(B)** and Cat **(D)** for 10 cluster. **(E)** The enriched GO terms of cluster 10 are shown.
