# Supplementary figures and images for: Single-Cell Sequencing Analysis of the db/db Mouse Hippocampus Reveals Cell-Type-Specific Insights Into the Pathobiology of Diabetes-Associated Cognitive Dysfunction
Source: Front Endocrinol (Lausanne). 2022 Jun 1;13:891039. doi: 10.3389/fendo.2022.891039 (PMC9200615; doi:10.3389/fendo.2022.891039)

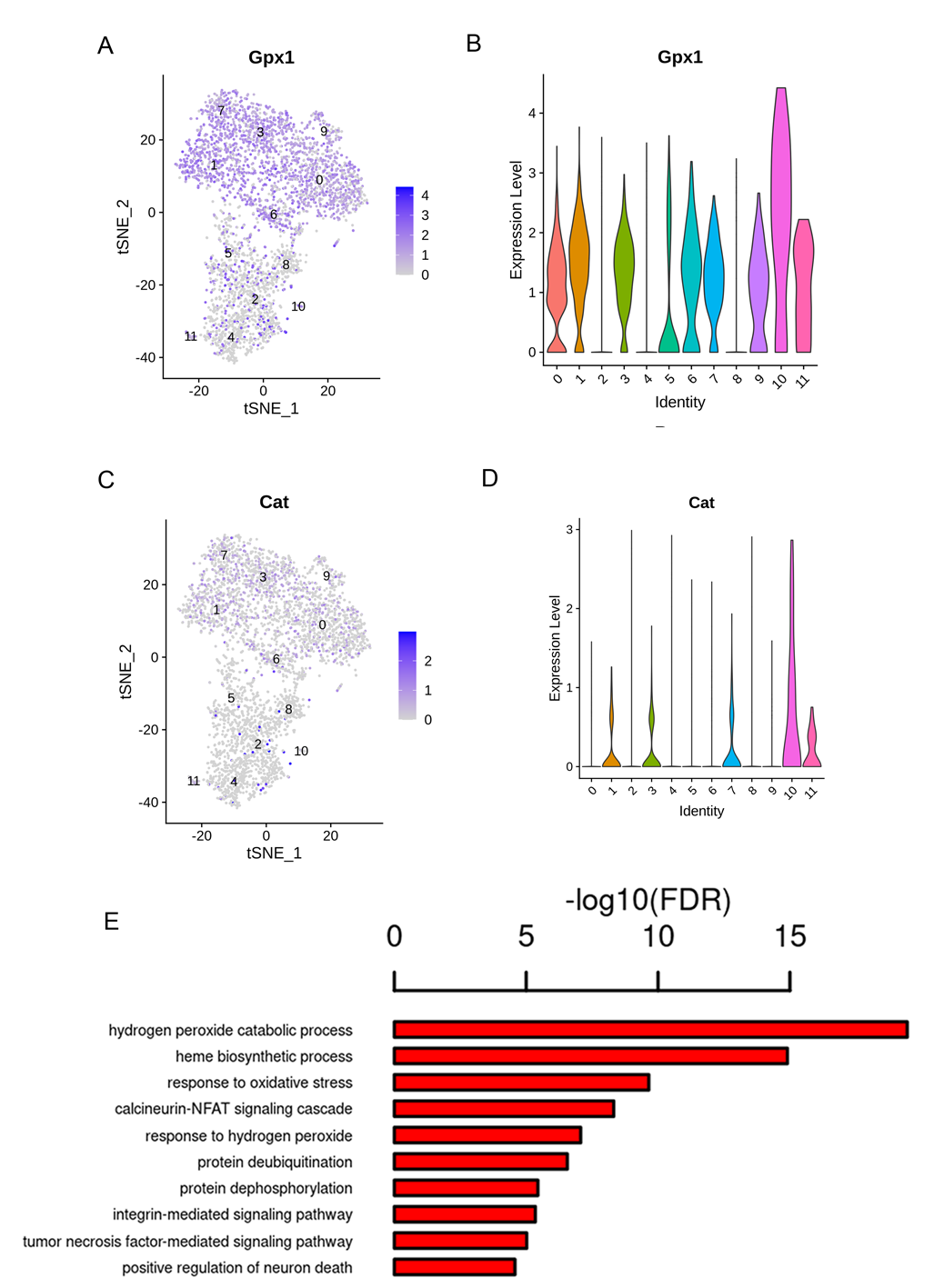

Supplement: Supplementary Figure 1 — Gene expression pattern analyses in cluster 10. Feature plot of inflammation marker genes Gpx1 (A) and Cat (C) in cluster 10. Violin plots showing the expression of inflammation marker genes Gpx1 (B) and Cat (D) for 10 cluster. (E) The enriched GO terms of cluster 10 are shown. [file Image_1.tif]
